# The predictive value of SOFA and APSIII scores for 28-day mortality risk in SIMI: a cohort study based on the MIMIC-IV database

**DOI:** 10.3389/fcimb.2025.1574625

**Published:** 2025-07-29

**Authors:** Chang Liu, Hao Wang, Chenyang Liu, Min Cao

**Affiliations:** Longhua Hospital, Shanghai University of Traditional Chinese Medicine, Shanghai, China

**Keywords:** sepsis, sepsis-induced myocardial injury (SIMI), SOFA score, APSIII score, mortality risk, MIMIC-IV database

## Abstract

**Objective:**

The objective of this study was to systematically identify and evaluate scoring systems that predict the prognosis of patients with sepsis-induced myocardial injury (SIMI).

**Methods:**

Data were retrieved from the Medical Information Mart for Intensive Care IV (MIMIC-IV) database. Logistic and Cox regression analyses were conducted to identify risk factors associated with 28-day mortality in patients with sepsis-induced myocardial injury (SIMI). The prognostic performance of the scoring systems was comprehensively assessed using receiver operating characteristic (ROC) curves, Kaplan-Meier survival and decision curve analysis (DCA).

**Results:**

Logistic regression analysis showed that Acute Physiology Score III (APSIII) (Odds Ratio [OR] =1.011, 95% Confidence Interval [CI] 1.002–1.018, P=0.005), Sequential Organ Failure Assessment (SOFA) (OR =1.097, 95% CI 1.045–1.144, P<0.001), and Charlson Comorbidity Index (CHARLSON) (OR=1.095, 95% CI 1.048–1.145, P=0.036) were independent risk factors for 28-day mortality in SIMI patients. Cox regression analysis confirmed that SOFA (HR=1.082, 95% CI 1.054– 1.111, P<0.001), APSIII (HR=1.010, 95% CI 1.005–1.015, P<0.001), and CHARLSON (HR=1.044, 95% CI 1.012–1.077, P=0.007) were independent risk factors. ROC curve analysis showed that SOFA (AUC=0.685, 95% CI 0.663–0.707) and APSIII (AUC=0.683, 95% CI 0.662–0.705) had significantly higher AUC values compared to other scoring systems. DCA results showed that APSIII and SOFA had better net benefit than other scoring systems.

**Conclusions:**

The SOFA and APSIII scores effectively identified high-risk patients with SIMI, providing evidence-based support for early clinical intervention.

## Introduction

Sepsis is a dysregulated inflammatory response to infection that can lead to life-threatening organ dysfunction. It is one of the leading causes of morbidity and mortality in intensive care units (ICUs). According to the Global Burden of Disease (GBD) study, sepsis has a global age-standardized incidence of 677.5 cases per 100,000 population (95% uncertainty interval [UI] 535.7–876.1), an age-standardized mortality rate of 148.1 deaths per 100,000 (95% UI 136.4–161.0), and accounts for 19.7% of all global deaths (Rudd et al., 2020). Among the organs affected by sepsis, the heart is particularly vulnerable, with sepsis-induced myocardial injury (SIMI) being a significant and often underrecognized complication (Narváez et al., 2018). SIMI typically manifests as left ventricular systolic or diastolic dysfunction, or both. The incidence of SIMI varies widely, ranging from 13.8% to 51.6%, depending on the study population and diagnostic criteria (Sato et al., 2016; Lu et al., 2024). This variability underscores the complexity and heterogeneity of SIMI. Despite its high incidence, SIMI is frequently underestimated and undertreated due to the lack of specific clinical symptoms and the absence of a gold standard diagnostic test (Kim et al., 2020). Recent studies have highlighted the profound impact of SIMI on patient outcomes. A prospective cohort study conducted in the Netherlands revealed that among patients with SIMI, the in-hospital mortality rate was 35%, while the 1-year mortality rate reached 51%, which further demonstrated a strong correlation between SIMI and increased mortality (Frencken et al., 2018). Additionally, SIMI has been associated with prolonged ICU stays and an elevated risk of long-term cardiovascular complications (Landesberg et al., 2012). Given these findings, early identification and appropriate management of SIMI are crucial for improving patient outcomes.

The clinical diagnosis of SIMI is highly challenging. Traditional echocardiographic parameters, such as left ventricular ejection fraction (LVEF), have notable limitations in detecting early myocardial dysfunction and predicting outcomes in sepsis patients (Sevilla Berrios et al., 2014). Moreover, single biomarkers such as troponin and B-type natriuretic peptide (BNP) are often nonspecific and can be influenced by a variety of factors (Sanfilippo et al., 2015). Therefore, there is a critical need for more reliable and comprehensive diagnostic tools to accurately identify SIMI.

Scoring systems have been extensively employed in critical care to assess disease severity and predict patient outcomes. Commonly used scoring systems include the SOFA, the Acute Physiology and Chronic Health (APACHE), and the Simplified Acute Physiology Score (SAPS) (Qian et al., 2024). These systems integrate multiple physiological parameters to provide a holistic evaluation of multi-system function in critically ill patients. However, previous research has predominantly focused on the general sepsis population, with a notable gap in predictive models specifically targeting the high-risk subgroup of SIMI. Given the limitations of existing diagnostic tools and the potential benefits of scoring systems, this study systematically evaluates the scoring efficacy for SIMI patients in the MIMIC-IV database, assessing their predictive value for 28-day mortality in SIMI patients and providing evidence-based support for risk stratification and clinical decision-making.

## Methods

### Data source

All data were sourced from the MIMIC-IV database, a large-scale public database developed by the Massachusetts Institute of Technology (MIT). This database encompasses comprehensive clinical data from patients treated in the ICU of Beth Israel Deaconess Medical Center between 2008 and 2019, including admission assessments, laboratory tests, therapeutic interventions, and other critical care-related information. To access the database, we completed the required training on the National Institutes of Health (NIH) website and signed a data use agreement. This study strictly adhered to the Health Insurance Portability and Accountability Act (HIPAA) to ensure the legality of data use and the protection of patient privacy. Moreover, the study methods and reporting of results followed the guidelines of the Transparent Reporting of a Multivariable Prediction Model for Individual Prognosis or Diagnosis (TRIPOD) statement, ensuring the transparency and scientific rigor of the research.

### Study population

**Figure 1:**
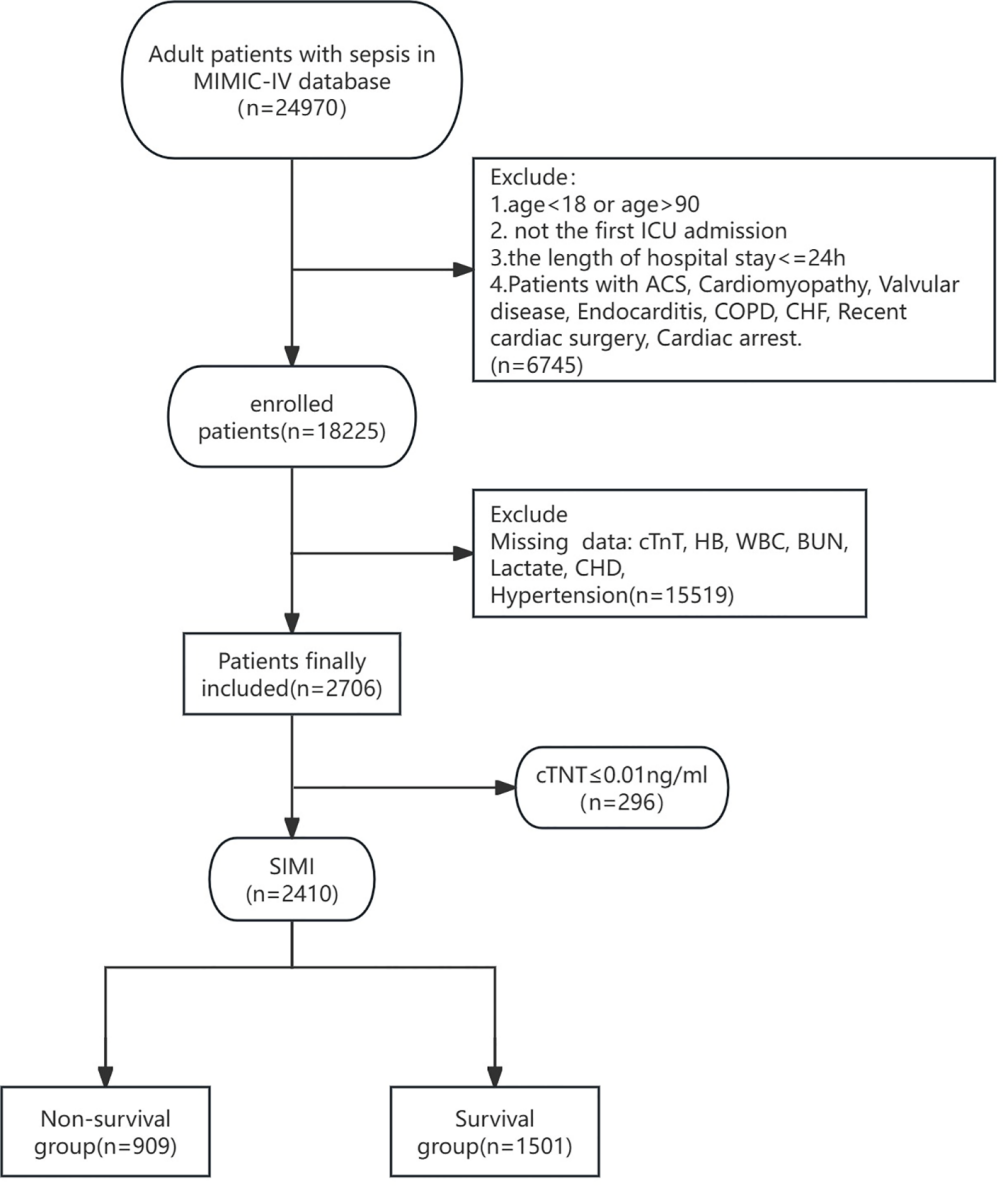
Flow diagram of this research. ICU, Intensive Care Unit; ACS, Acute Coronary Syndrome; COPD, Chronic Obstructive Pulmonary Disease; CHF, Chronic Heart Failure; cTnT, cardiac troponin T; HB, Hemoglobin; WBC, White Blood Cell Count; BUN, Blood Urea Nitrogen; CHD, Coronary Heart Disease.

All adult patients diagnosed with SIMI (first admission only) were extracted from the MIMIC-IV database. Inclusion criteria included: (1) meeting the diagnostic criteria for sepsis 3.0 with the SOFA score ≥2; (2) meeting the diagnostic criteria for SIMI; (3) admission to the ICU with age ≥18 years; (4) ICU length of stay >24 hours but not exceeding 100 days. Exclusion criteria included: (1) SOFA score <2 and cardiac troponin T (cTnT) <0.01 ng/mL; (2) exclusion of patients with missing baseline data, such as unrecorded lactate, hemoglobin (Hb), international normalized ratio (INR), white blood cell count (WBC), blood urea nitrogen (BUN), blood pressure (BP), temperature (T), respiratory rate (RR), etc.; (3) exclusion of direct or indirect causes of abnormal release of cTnT, including acute coronary syndrome (ACS), cardiomyopathy, myocarditis, valvular disease, endocarditis, pericarditis, chronic obstructive pulmonary disease (COPD), chronic heart failure (CHF), history of cardiac surgery or cardiac arrest prior to ICU admission, and history of significant tachyarrhythmias (e.g., supraventricular tachycardia, ventricular tachycardia, ventricular fibrillation, ventricular flutter) (**Figure 1**).

### Data extraction and management

Data extraction was conducted using Navicat Premium software (version 15.0) from the MIMIC-IV database. The extracted data comprised demographic characteristics such as age (recorded in years), gender (categorized as male or female), and race (self-reported racial identification). Clinical information encompassed RR (measured in breaths per minute), BP (recorded as systolic and diastolic pressures in mmHg), HR (measured in beats per minute), oxygen saturation (SpO_2_, measured as a percentage), body temperature (recorded in degrees Celsius), comorbid conditions (including hypertension, diabetes mellitus, hyperlipidemia, coronary artery disease [CAD], and chronic kidney disease), source of infection (categorized based on clinical diagnosis), and critical care scoring systems (SOFA, APSIII, Systemic Inflammatory Response Syndrome [SIRS], Logistic Organ Dysfunction System [LODS], Outcome Prediction in Acute Physiology and Age [OASIS], CHARLSON, Glasgow Coma Scale [GCS], and Model for End-Stage Liver Disease [MELD]). Laboratory parameters included hematocrit (HCT, measured as a percentage), platelet count (PLT, measured in 10^9/L), hemoglobin level (HB, measured in g/dL), white blood cell count (WBC, measured in 10^9/L), prothrombin time (PT, measured in seconds), serum creatinine (Cr, measured in mg/dL), BUN (measured in mg/dL), INR (measured as a ratio), and serum lactate (measured in mmol/L). For laboratory tests and vital signs recorded multiple times within the first 24 hours of hospitalization, median values were used to represent the initial clinical status, thereby minimizing the impact of transient fluctuations and providing a more stable baseline for analysis.

### SIMI criteria

SIMI refers to the myocardial dysfunction that occurs in patients with sepsis, characterized by reversible myocardial depression. The diagnostic criteria for SIMI are primarily based on the levels of cTnT. According to the recommendations of the International Federation of Clinical Chemistry and Laboratory Medicine (IFCC), the 99th percentile upper reference limit for cTnT is 0.01 ng/mL, Therefore, SIMI is defined as a cTnT level exceeding 0.01 ng/mL (Vallabhajosyula et al., 2017; Xu et al., 2023; Xie et al., 2021). On the day of ICU admission, cTnT levels were measured, and the highest value obtained on that day was used for SIMI assessment.

### Statistical analysis

Normally distributed continuous data were expressed as mean ± standard deviation, while categorical variables were summarized using counts and percentages. Comparisons between groups for categorical variables were performed using chi-square tests or Fisher’s exact tests, as appropriate. Student’s t-tests were used for comparisons of continuous variables between two groups. Univariate analysis was conducted to identify potential risk factors for in-hospital mortality, with variables having a p-value of less than 0.05 included in the subsequent multivariate logistic regression and Cox regression analyses analysis to determine independent risk factors for hospital death. Logistic regression analysis was conducted using built-in functions, while Cox regression analysis was implemented using the “survival” package in R software (Therneau, 2024). The predictive capacity of each scoring system for mortality was evaluated by comparing ROC curves and the area under the ROC curves (AUC). Optimal cut-off values were derived from the ROC curves, and Kaplan-Meier survival curves were constructed to assess the prognostic impact of the scoring systems on survival outcomes in high-risk and low-risk patient subgroups. ROC curves were generated using the “pROC” package in R (Robin et al., 2011). DCA was applied to assess the net benefit of the scoring systems for patients, with a larger area under the DCA curve indicating greater clinical utility. DCA was performed using the “rmda” package in R (Brown, 2018). All statistical analyses were performed using R software (version 4.2.0, R Foundation for Statistical Computing), with a two-sided p-value of less than 0.05 considered to indicate statistical significance.

## Results

### Baseline characteristics

A total of 18,225 patient records were harvested from the MIMIC-IV database. After excluding patients who did not meet the diagnostic criteria for Sepsis 3.0, those with missing data, and those who did not meet the SIMI definition, a final cohort of 2,310 patients was included in the analysis. Patients were divided into two groups based on 28-day all-cause mortality: the survival group (n=1,501) and the non-survival group (n=909). Comparison of baseline characteristics revealed that the non-survival group had a higher mean age (69.93 years vs. 66.41 years, P<0.001) and a higher proportion of patients requiring mechanical ventilation (83.9% vs. 74.1%, P<0.001). Also, the non-survival group had a higher vasopressin use rate (13.3% vs. 8.3%, p<0.01). But the two groups had no difference in fluid balance. Additionally, the non-survival group had significantly higher scores on the SOFA, APS III, SIRS, LODS, OASIS, Charlson Comorbidity Index, and MELD scales. Other variables that showed significant differences between the two groups included WBC, HB, BUN, Cr, INR, lactate, oxygenation index, T, RR, HR, mean arterial pressure (MAP), hypertension, and liver disease. There were no significant differences between the two groups in terms of CHD, diabetes, and chronic kidney disease. However, the survival group had a higher blood glucose level than the non-survival group (158.2 vs 171.54, p<0.05). (**Table 1**).

**Table 1:** Baseline characteristics.

Variables	Non-survival	Survival	P
SIMI patients, n(%)	909 (37.7%)	1501 (62.3%)	
Demographics			
Age (years)	69.93 (68.93, 70.94)	66.41 (65.62, 67.19)	<0.001
Male (%)	539 (60.7%)	911 (60.7%)	0.525
LOS Hos (days)	11.39 (10.56, 12.22)	19.59 (18.83, 20.36)	<0.001
LOS ICU (days)	6.80 (6.35, 7.25)	7.77 (7.36, 8.17)	0.002
White (%)	474 (52.1%)	878 (58.5%)	<0.001
Life-support			
vasopressin (%)	121 (13.3%)	124 (8.3%)	<0.001
Mechanical ventilation (%)	763 (83.9%)	1112 (74.1%)	<0.001
Laboratory tests			
WBC (109/L)	16.36 (15.58, 17.15)	14.74 (14.07, 15.41)	0.002
HB (g/dl)	10.76 (10.59, 10.92)	10.98 (10.85, 11.10)	0.037
NT-proBNP (pg/mL)	11778.38 (9625.31, 13931.46)	11849.49 (9963.74, 13735.23)	0.961
PLT (109/L)	202.11 (194.62, 209.60)	209.70 (204.29, 215.11)	0.107
BUN (mmol/L)	42.58 (40.56, 44.60)	34.28 (32.93, 35.63)	<0.001
Cr (mg/dl)	2.18 (2.07, 2.29)	1.99 (1.89, 2.09)	0.014
INR	1.83 (1.74, 1.91)	1.55 (1.50, 1.60)	0.015
Lactate (mmol/L)	4.03 (3.79, 4.27)	2.51 (2.40, 2.62)	<0.001
TG (mg/dL)	185.02 (154.73, 215.30)	178.35 (161.46, 195.24)	0.705
HDL (mg/dL)	41.79 (38.05, 45.53)	43.89 (40.71, 47.07)	0.398
LDL (mg/dL)	78.72 (69.81, 87.64)	76.94 (69.71, 84.17)	0.758
CRP (mg/L)	130.29 (107.82, 152.77)	111.49 (90.69, 132.29)	0.223
BG (mg/dL)	158.20 (155.36, 161.05)	171.54 (166.46, 176.62)	<0.001
PaO2/FiO2 (mmHg)	238.04 (227.24, 248.85)	252.52 (243.90, 261.14)	0.040
Vital signs			
T (°C)	33.77 (33.14, 34.39)	35.63 (35.31, 35.96)	<0.001
HR (bmp)	96.46 (94.94, 97.98)	93.65 (92.51, 94.80)	0.004
RR (cmp)	22.40 (21.97, 22.83)	21.31 (21.00, 21.62)	<0.001
MAP (mmhg)	81.50 (80.13, 82.87)	83.92 (82.85, 84.98)	0.006
Fluid balance (mL)	-19.31 (-47.35, 8.72)	-17.44 (-33.69, -1.19)	0.910
Coexisting comorbidities			
Hypertension	203 (22.3%)	426 (28.4%)	0.001
Coronary atherosclerotic heart disease	49 (5.4%)	94 (6.3%)	0.430
Liver disease	103 (11.3%)	73 (4.9%)	<0.001
Diabetes	311 (34.2%)	549 (36.6)	0.259
Chronic kidney disease	263 (28.9%)	448 (29.8%)	0.667
Scoring systems			
SOFA	11.13 (10.85, 11.40)	8.40 (8.21, 8.58)	<0.001
APSIII	73.12 (71.42, 74.82)	56.92 (55.83, 58.01)	<0.001
GCS	9.49 (9.17, 9.82)	10.91 (10.71, 11.11)	<0.001
SIRS	3.17 (3.12, 3.22)	2.96 (2.92, 3.00)	<0.001
LODS	8.42 (8.21, 8.62)	6.57 (6.42, 6.72)	<0.001
CHARLSON	6.32 (6.12, 6.52)	5.44 (5.30, 5.59)	<0.001
MELD	22.37 (21.76, 22.98)	18.15 (17.74, 18.57)	<0.001
OASIS	41.47 (40.92, 42.02)	36.95 (36.54, 37.37)	<0.001

T, Temperature; HR, Heart Rate; RR, Respiratory Rate; MAP, Mean Arterial Pressure; WBC, White Blood Cell Count; HB, Hemoglobin; NT-proBNP, N-Terminal Pro-B-Type Natriuretic Peptide; PLT, Platelet Count; BUN, Blood Urea Nitrogen; Cr, Creatinine; INR, International Normalized Ratio; TG, Triglycerides; HDL, High-Density Lipoprotein; LDL, Low-Density Lipoprotein; CRP, C-Reactive Protein; LOS Hos, Length of Stay in Hospital; LOS ICU, Length of Stay in Intensive Care Unit; CHARLSON, Charlson Comorbidity Index; SOFA, Sequential Organ Failure Assessment; APSIII, Acute Physiology Score III; GCS, Glasgow Coma Scale; SIRS, Systemic Inflammatory Response Syndrome; LODS, Logistic Organ Dysfunction System; MELD, Model for End-Stage Liver Disease; OASIS, Oxford Acute Severity of Illness Score.

### Logistic regression analysis

Multivariate logistic regression analysis was conducted to identify independent risk factors for 28-day mortality among patients with SIMI. The analysis revealed that age (OR = 1.017, 95% CI 1.008–1.026), serum lactate levels (OR = 1.145, 95% CI 1.102–1.190),serum BUN level (OR = 1.004, 95% CI 1.000–1.008), liver disease (OR = 1.491, 95% CI 1.019–2.185), APSIII (OR = 1.011, 95% CI 1.002–1.018), SOFA (OR = 1.097, 95% CI 1.045–1.144), and CHARLSON (OR = 1.095, 95% CI 1.048–1.145) were independently associated with increased risk of 28-day mortality. Conversely, diabetes mellitus (OR = 0.677, 95% CI 0.546-0.839), BG (OR=1.002 95%CI 1.000-1.003) and GCS (OR = 0.975, 95% CI 0.952-0.999) were identified as protective factors against mortality (**Table 2**).

**Table 2:** Logistic regression analysis.

Variable	Univariate analysis		Multivariate analysis	
	P	OR (95%)	P	OR (95%)
SOFA	<0.001	1.196 (1.169, 1.224)	<0.001	1.098 (1.058, 1.139)
APSIII	<0.001	1.029 (1.025, 1.033)	0.008	1.010 (1.003, 1.018)
GCS	<0.001	0.930 (0.912, 0.947)	0.042	0.975 (0.952, 0.999)
SIRS	<0.001	1.365 (1.232, 1.515)	0.068	1.121 (0.992, 1.269)
LODS	<0.001	1.216 (1.182, 1.252)	0.842	1.005 (0.957, 1.056)
CHARLSON	<0.001	1.105 (1.074, 1.136)	<0.001	1.095 (1.048, 1.145)
MELD	<0.001	1.056 (1.046, 1.066)	0.440	0.994 (0.979, 1.009)
OASIS	<0.001	1.067 (1.056, 1.079)	0.911	0.992 (0.982, 1.017)
Age (year)	<0.001	1.015 (1.010, 1.021)	<0.001	1.018 (1.009, 1.026)
HB (g/dl)	0.036	0.965 (0.934, 0.998)	0.550	1.012 (0.973, 1.052)
WBC (109/L)	0.005	1.011 (1.004, 1.079)	0.368	1.003 (0.996, 1.011)
BUN (mmol/L)	<0.001	1.010 (1.007,1.013)	0.050	1.004 (1.000, 1.007)
Lactate (mmol/L)	<0.001	1.209 (1.171, 1.251)	<0.001	1.134 (1.091, 1.180)
BG (mg/dL)	<0.001	1.003 (1.002, 1.004)	0.040	1.002 (1.000, 1.003)
Hypertension	0.001	0.726 (0.598, 0.878)	0.329	0.898 (0.724, 1.113)
Coronary atherosclerotic heart disease	0.380	0.853 (0.594, 1.211)	0.617	0.913 (0.616, 1.341)
Liver disease	<0.001	2.500 (1.833, 3.425)	0.032	1.518 (1.038, 2.225)
Diabetes	0.241	0.902 (0.758, 1.071)	<0.001	0.617 (0.489, 0.775)
Mechanical ventilation (%)	<0.001	1.828 (1.483, 2.264)	0.197	1.240 (0.905, 1.634)
Vasopressin (%)	<0.001	1.705 (1.307, 2.224)	0.685	1.063 (0789, 1.430)

CHARLSON, Charlson Comorbidity Index; SOFA, Sequential Organ Failure Assessment; APSIII, Acute Physiology Score III; GCS, Glasgow Coma Scale; SIRS, Systemic Inflammatory Response Syndrome; LODS, Logistic Organ Dysfunction System; MELD, Model for End-Stage Liver Disease; OASIS, Oxford Acute Severity of Illness Score; HB, Hemoglobin; WBC, White Blood Cell Count; BUN, Blood Urea Nitrogen.

### Cox regression analysis

Cox proportional hazards regression analysis was conducted to identify independent risk factors for 28-day mortality among patients with SIMI. The analysis revealed that age (HR = 1.014, 95% CI 1.008–1.020), serum lactate levels (HR = 1.073, 95% CI 1.052–1.094), SOFA (HR = 1.082, 95% CI 1.054–1.115), APSIII (HR = 1.010, 95% CI 1.005–1.015), and CHARLSON (HR = 1.044, 95% CI 1.012–1.077) were independent risk factors for the 28-day risk of death. Conversely, diabetes mellitus (HR = 0.747, 95% CI 0.641–0.871) and BG (HR=1.003 95%CI 1.002-1.004) was protective factor. These data were consistent with the results from the logistic regression analysis, further validating the identified risk factors and protective factors (**Table 3**).

**Table 3:** Cox regression analysis.

Variable	Univariate analysis		Multivariate analysis	
	P	HR(95%)	P	HR(95%)
SOFA	<0.001	1.147 (1.129, 1.165)	<0.001	1.085 (1.056, 1.113)
APSIII	<0.001	1.022 (1.020, 1.025)	<0.001	1.010 (1.004, 1.015)
GCS	<0.001	0.946 (0.932, 0.960)	0.267	1.010 (0.992, 1.028)
SIRS	<0.001	1.295 (1.193, 1.406)	0.198	1.062 (0.969, 1.163)
LODS	<0.001	1.166 (1.143, 1.189)	0.750	1.006 (0.971, 1.042)
CHARLSON	<0.001	1.071 (1.048, 1.094)	0.003	1.047 (1.016, 1.080)
MELD	<0.001	1.045 (1.037, 1.052)	0.638	0.824 (0.987, 1.008)
OASIS	<0.001	1.056 (1.048, 1.094)	0.786	0.999 (0.988, 1.009)
Age (year)	<0.001	1.011 (1.007, 1.016)	<0.001	1.015 (1.009, 1.021)
HB (g/dl)	0.217	0.984 (0.958, 1.010)	0.026	1.031 (1.004, 1.061)
WBC (109/L)	0.008	1.004 (1.001, 1.007)	0.959	1.000 (0.996, 1.004)
BUN (mmol/L)	<0.001	1.007 (1.005, 1.009)	0.093	1.002 (1.000, 1.004)
Lactate (mmol/L)	<0.001	1.128 (1.111, 1.146)	<0.001	1.067 (1.046, 1.088)
BG (mg/dL)	<0.001	1.003 (1.002, 1.004)	<0.001	1.003 (1.002, 1.004)
Hypertension	0.002	0.781 (0.668, 0.913)	0.657	0.965 (0.822, 1.132)
Coronary atherosclerotic heart disease	0.362	0.875 (0.656, 1.166)	0.564	0.918 (0.686, 1.228)
Liver disease	<0.001	1.767 (1.440, 2.170)	0.305	1.132 (0.893, 1.434)
Diabetes	0.154	0.905 (0.789, 1.038)	<0.001	0.649 (0.550, 0.766)
Mechanical ventilation (%)	<0.001	1.709 (1.432, 2.040)	0.199	1.160 (0.925, 1.455)
Vasopressin (%)	<0.001	1.469 (1.213, 1.779)	0.680	1.054 (0.857, 1.267)

CHARLSON, Charlson Comorbidity Index; SOFA, Sequential Organ Failure Assessment; APSIII, Acute Physiology Score III; GCS, Glasgow Coma Scale; SIRS, Systemic Inflammatory Response Syndrome; LODS, Logistic Organ Dysfunction System; MELD, Model for End-Stage Liver Disease; OASIS, Oxford Acute Severity of Illness Score; HB, Hemoglobin; WBC, White Blood Cell Count; BUN, Blood Urea Nitrogen.

### Comparison of receiver operating characteristic curves for scoring systems

ROC curve analysis was conducted to evaluate and compare the predictive accuracy of various scoring systems for 28-day mortality in patients with SIMI. The AUC was used as a quantitative index to measure the discrimination ability of each scoring system between survivors and non-survivors. The results indicated that the AUC values for the SOFA (AUC = 0.685, 95% CI 0.663–0.707), APS III (AUC = 0.683, 95% CI 0.662–0.705), LODS (AUC = 0.678, 95% CI 0.655–0.701), OASIS (AUC = 0.669, 95% CI 0.646–0.692), and MELD (AUC = 0.654, 95% CI 0.632–0.676) scoring systems were all above 0.6, suggesting acceptable predictive performance. Notably, the SOFA and APS III scoring systems demonstrated superior predictive accuracy, with AUC values significantly higher than those of the other systems.

**Figure 2:**
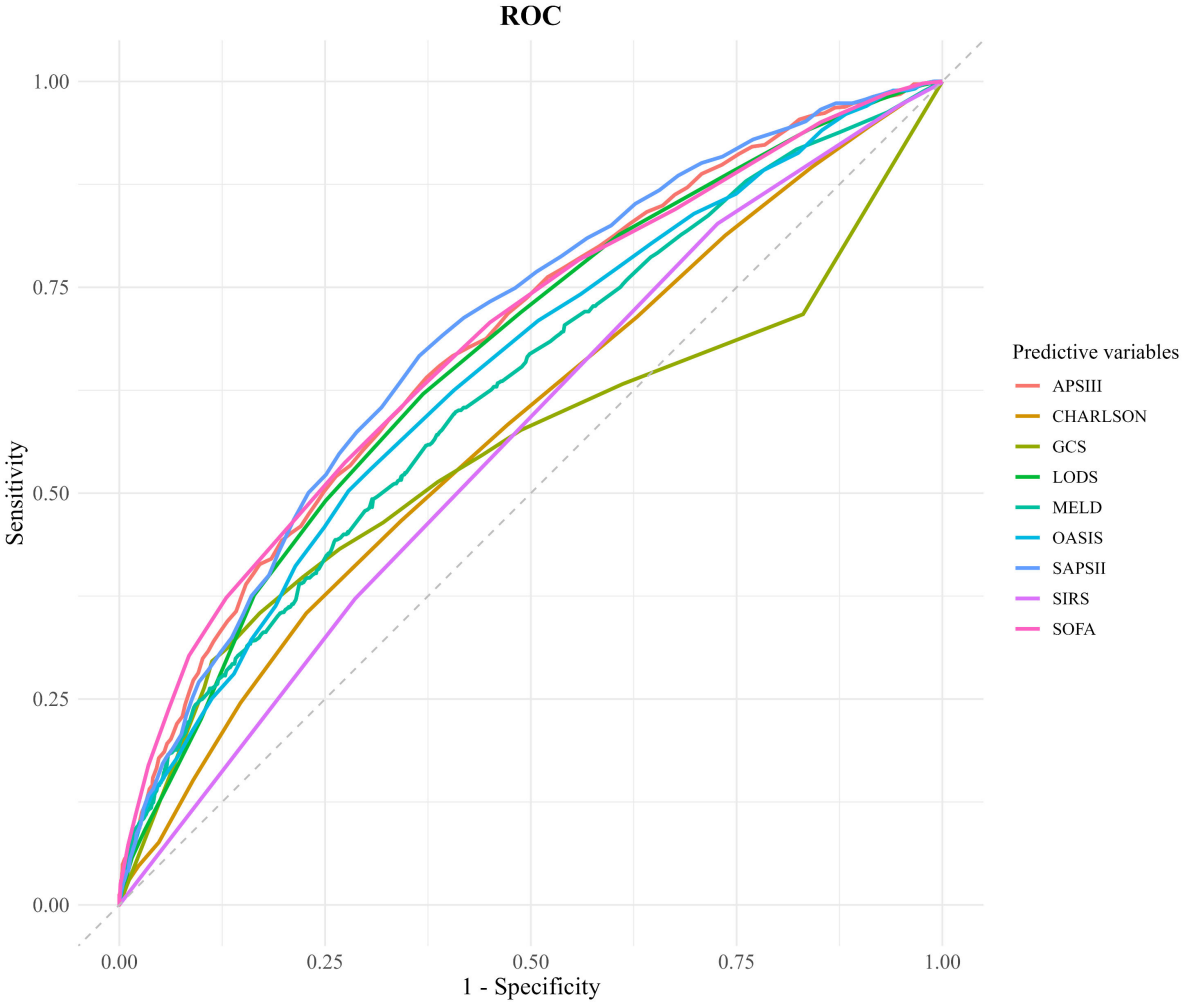
Comparison of decision curve analysis curve. CHARLSON, Charlson Comorbidity Index; SOFA, Sequential Organ Failure Assessment; APSIII, Acute Physiology Score III; GCS, Glasgow Coma Scale; SIRS, Systemic Inflammatory Response Syndrome; LODS, Logistic Organ Dysfunction System; MELD, Model for End-Stage Liver Disease; OASIS, Oxford Acute Severity of Illness Score.

To identify the optimal cut-off points for each scoring system, the Youden index was calculated. For the APS III scoring system, the Youden index was 0.267, corresponding to a sensitivity of 64% and a specificity of 62.7%. For the SOFA scoring system, the Youden index was 0.263, with a sensitivity of 53.7% and a specificity of 72.6%. These findings highlight the effectiveness of both APS III and SOFA scoring systems in predicting in-hospital mortality, offering a balanced trade-off between sensitivity and specificity. The ROC curves for these scoring systems are depicted in **Figure 2** and **Table 4**.

**Table 4:** Diagnostic performance of scoring systems.

Predictor	AUC	95%Cl	Optimal cut-of	Sensitivity	Specificity
SOFA	0.685	(0.663, 0.707)	10.5	0.537	0.726
APSIII	0.683	(0.662, 0.705)	61.5	0.64	0.627
GCS	0.552	(0.527, 0.578)	6.5	0.354	0.829
SIRS	0.567	(0.546, 0.589)	2.5	0.827	0.273
LODS	0.667	(0.645, 0.689)	7.5	0.62	0.631
CHARLSON	0.582	(0.558, 0.605)	7.5	0.354	0.773
MELD	0.629	(0.606, 0.652)	19.573	0.598	0.592
OASIS	0.647	(0.624, 0.669)	41.5	0.503	0.722

CHARLSON, Charlson Comorbidity Index; SOFA, Sequential Organ Failure Assessment; APSIII, Acute Physiology Score III; GCS, Glasgow Coma Scale; SIRS, Systemic Inflammatory Response Syndrome; LODS, Logistic Organ Dysfunction System; MELD, Model for End-Stage Liver Disease; OASIS, Oxford Acute Severity of Illness Score; AUC, Area Under the Curve.

### Kaplan-meier survival analysis for APS III and SOFA scoring systems

Utilizing the optimal cut-off values derived from the ROC curve analysis and Youden index calculations, patients were stratified into high-risk and low-risk subgroups based on their APS III and SOF. Specifically, for the APS III score, the ideal cut-off value was determined to be 61.5. Patients with scores above this threshold were classified into the high-risk subgroup, whereas those with scores below were categorized into the low-risk subgroup. Kaplan-Meier survival analysis revealed that patients in the high-risk APS III subgroup had a significantly higher hazard ratio (HR = 2.454, 95% CI 2.143–2.811) compared to their low-risk counterparts, indicating a markedly poorer prognosis.

Similarly, for the SOFA score, the optimal cut-off value was identified as 10.5. Patients were again divided into high-risk and low-risk subgroups based on this threshold. The Kaplan-Meier analysis demonstrated that patients in the high-risk SOFA subgroup exhibited significantly higher 28-day mortality compared to those in the low-risk subgroup (HR = 2.445, 95% CI 2.145–2.785) further emphasizing the prognostic significance of the SOFA score in predicting adverse outcomes.

**Figure 3:**
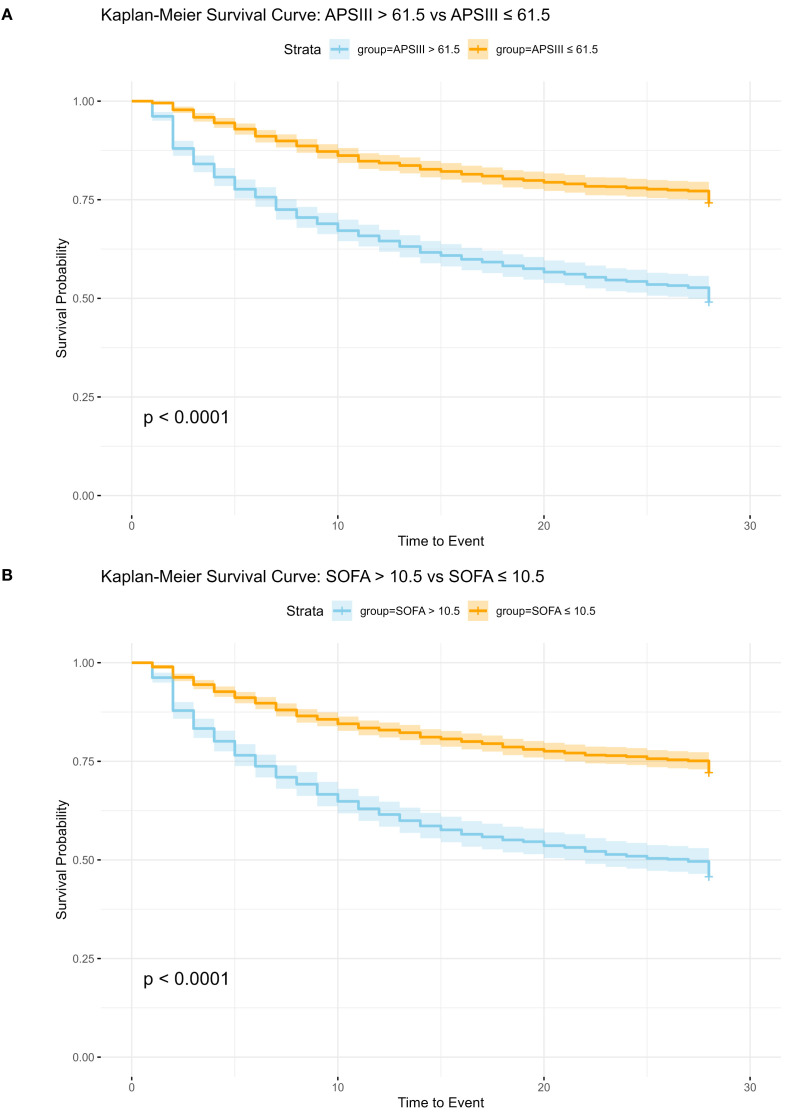
Kaplan-Meier Survival Analysis for APS III and SOFA Scoring Systems. SOFA, Sequential Organ Failure Assessment; APSIII, Acute Physiology Score III; **(A)** Kaplan-Meier Survival Analysis for APS III, HR: 2.454 (95%CI 2.143, 2.811 p<0.01). **(B)** Kaplan-Meier Survival Analysis for SOFA, HR: 2.445 (95%CI 2.145, 2.785 p<0.01).

These results suggested the utility of both APS III and SOFA scores as robust predictors of in-hospital mortality, with Kaplan-Meier curves visually illustrating the pronounced survival differences between the high-risk and low-risk subgroups (**Figures 3a, b**).

### Comparison of decision curve analysis curves

**Figure 4:**
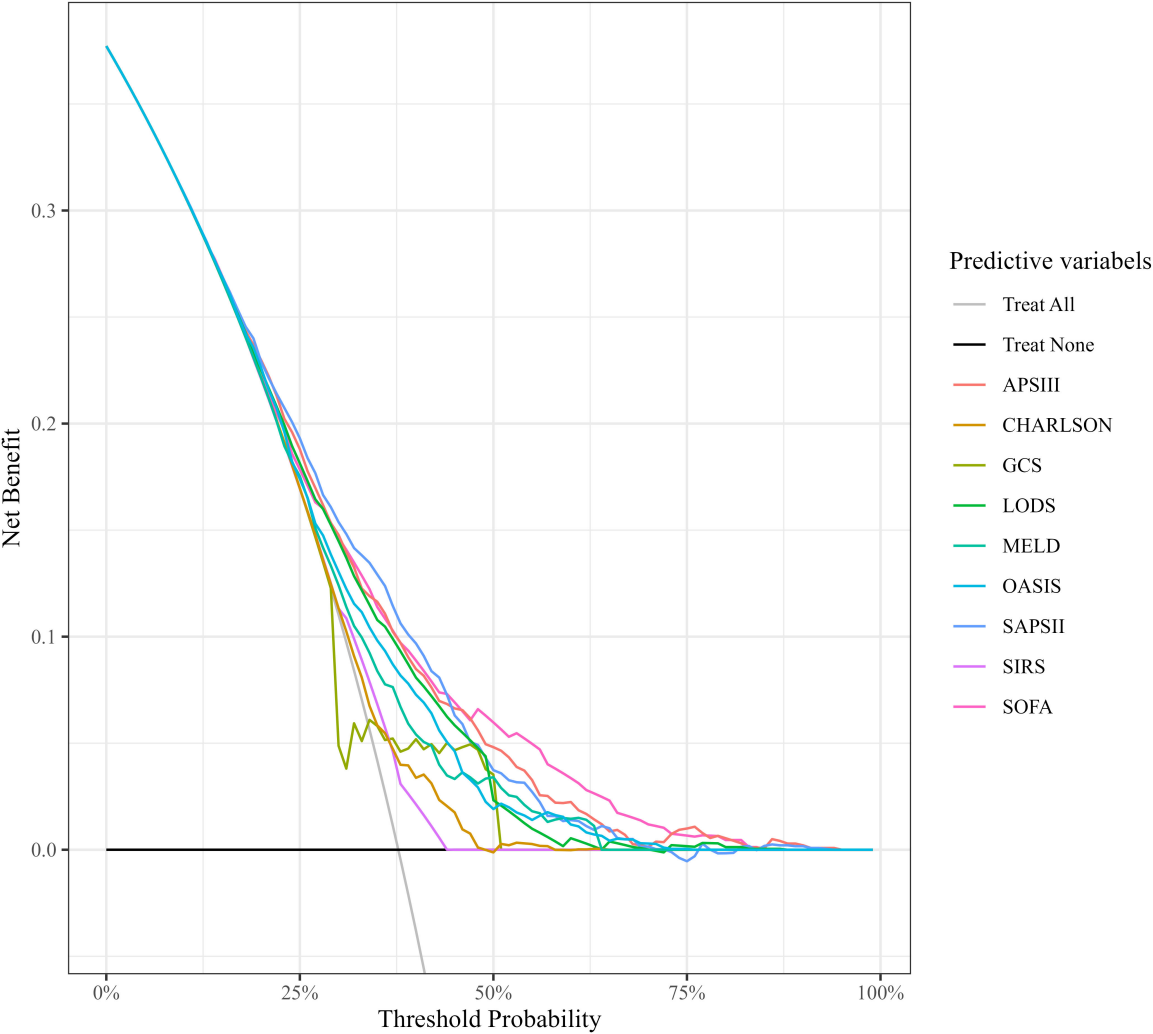
Comparison of decision curve analysis curves. CHARLSON, Charlson Comorbidity Index; SOFA, Sequential Organ Failure Assessment; APSIII, Acute Physiology Score III; GCS, Glasgow Coma Scale; SIRS, Systemic Inflammatory Response Syndrome; LODS, Logistic Organ Dysfunction System; MELD, Model for End-Stage Liver Disease; OASIS, Oxford Acute Severity of Illness Score.

The results of DCA indicated that the curves for the APS III and SOFA scoring systems consistently surpassed those of other scoring systems across the entire range of threshold probabilities. When the risk threshold was <20%, all scoring systems had benefits similar to “Treatment All” (i.e., treating all SIMI patients). But when the risk threshold was ≥20%, only SOFA and APSIII had higher benefits than both “Treatment All” and “Treatment None”. The analysis revealed that APS III and SOFA offer superior net clinical benefit compared to other systems, APS III and SOFA scores demonstrated higher net benefits, indicating that clinical interventions guided by these scores are likely to yield greater clinical utility and improved patient outcomes. These data illustrated the potential for enhanced clinical decision-making when utilizing APS III and SOFA scores for risk stratification and guiding interventions (**Figure 4**).

## Discussion

SIMI is characterized by ventricular systolic and/or diastolic dysfunction secondary to systemic inflammation and microcirculatory disturbances in sepsis patients, representing a frequent complication and major contributor to mortality in this population (de Souza et al., 2021). Although SIMI has been traditionally considered a transient condition with cardiac function recovery within 7–10 days in survivors (Hollenberg and Singer, 2021), emerging evidence confirms that myocardial injury remains an independent risk factor for adverse outcomes. A recent meta-analysis demonstrated a strong association between SIMI and mortality in patients hospitalized for more than 10 days (RR 1.40, 95% CI (1.02–1.93) (Lin et al., 2022). These investigations revealed the critical importance of early risk stratification, which reflected underlying pathophysiological mechanisms including mitochondrial energy metabolism abnormalities, calcium homeostasis imbalances, and inflammation-mediated myocardial depression in SIMI patients (Li et al., 2024). The development of timely assessment protocols and predictive models for septic cardiomyopathy therefore constitutes a critical step toward improving patient prognosis.

Echocardiography is an essential tool for assessing cardiac structure and function. However, static parameters such as LVEF have limitations in reflecting dynamic changes in cardiac function and are not ideal for predicting prognosis in cardiomyopathies diseases. For example, Sevilla Berrios et al. (2014). found that LVEF was neither a sensitive (52%) nor a specific (63%) predictor of 30-day mortality in patients with sepsis, with AUC of only 0.62. Additionally, Sanfilippo et al. (2015) demonstrated that left ventricular systolic dysfunction is not associated with patient mortality outcomes (relative risk, RR = 0.93; 95% confidence interval, 0.62–1.39). Within the context of these limitations, it is necessary to explore alternative prognostic indicators. Recently, several novel echocardiographic parameters have emerged as more sensitive indicators of the potential left ventricular dysfunction. Parameters such as left ventricular longitudinal strain (LVLS), mitral annular plane systolic excursion (MAPSE), and left ventricular longitudinal wall shortening fraction (LV-LWFS) have shown promise in identifying subtle changes in cardiac function that traditional measures may overlook (Nagueh et al., 2016). These parameters leverage the dynamic nature of cardiac function, providing a more comprehensive assessment of myocardial performance. Beyond echocardiography, biomarkers such as BNP and NT-proBNP have been widely utilized for prognostic assessment in heart failure patients. A retrospective study demonstrated that patients with SIMI who died during hospitalization had significantly higher levels of BNP, cTnI, PCT, and myoglobin compared to survivors. However, ROC curve analysis revealed that only PCT achieved an AUC greater than 0.6 (Qian et al., 2024). Frencken et al. (2018) found that elevated troponin levels were independently associated with mortality within 14 days among sepsis patients with myocardial injury; But this association did not persist beyond 14 days, regardless of whether troponin levels were mildly, moderately, or highly elevated. Brueckmann et al. (2005) reported that sepsis patients with NT-proBNP levels exceeding 1400 pmol/L had a 3.9-fold higher risk of death compared to those with lower levels (RR = 3.9; 95% CI, 1.6 to 9.7). However, the sensitivity of NT-proBNP at this cutoff was only 50%.

A growing body of research has unveiled several novel biomarkers, including fibroblast growth factor-21 (FGF-21) and growth differentiation factor-15 (GDF-15), sST2, Lp-PLA2, and Lipocalin 10, that hold potential value for the diagnosis and prognosis prediction of sepsis-induced myocardial injury (SIMI).Growth differentiation factor-15 (GDF-15), a member of the transforming growth factor-β (TGF-β) superfamily, has garnered attention due to its significant correlation with organ injury and the severity of sepsis. A prospective cohort study conducted by Li et al. demonstrated that GDF-15 exhibits robust diagnostic efficacy for sepsis, with an area under the receiver operating characteristic curve (AUC) of 0.821, specificity of 85.61%, sensitivity of 72.26%, and a 95% confidence interval (CI) ranging from 0.772 to 0.864. Furthermore, *in vitro* experiments revealed that GDF-15 can attenuate inflammatory responses by modulating macrophage function, inhibiting JAK1/STAT3 phosphorylation, and preventing NF-κB p65 nuclear translocation (Li et al., 2021). Fibroblast growth factor-21 (FGF-21) is a growth factor that plays a pivotal role in regulating lipid and glucose metabolism. Increased expression of FGF-21 has been shown to inactivate the TGF-β1-Smad2/3-MMP2/9 signaling pathway, thereby mitigating myocardial fibrosis, oxidative stress, and cell apoptosis (Ma et al., 2021). Notably, Li et al. discovered that FGF21 can serve as a prognostic indicator for sepsis patients. Their findings indicated that sepsis patients with FGF21 levels below 3,200 pg/ml have a significantly lower mortality rate compared to those with levels above 3,200 pg/ml (Li et al., 2018). In addition, elevated levels of FGF-21 have been confirmed as a potent predictor of major adverse cardiovascular events (MACE) in ST-segment elevation myocardial infarction (STEMI) patients following emergency percutaneous coronary intervention (PCI), with a HR of 2.011 and a 95% CI of 1.160 - 3.489 (Gu et al., 2021). These results collectively suggest that FGF-21 holds promise as a predictive biomarker for sepsis-induced myocardial injury (SIMI). Lipoprotein-associated phospholipase A2 (Lp-PLA2) has been implicated in promoting vascular inflammation-related diseases through its mediation of macrophage migration and activation. Inhibition of Lp-PLA2 has been shown to block the activation of the macrophage NLRP3 inflammasome, thereby preventing angiotensin II (Ang II)-induced cardiac inflammation and fibrosis (Lv et al., 2021). Persistent elevation of serum Lp-PLA2 concentration is often indicative of a poor prognosis in sepsis patients. A cohort study revealed that Lp-PLA2 concentration is associated with disease severity, with a higher survival rate observed when Lp-PLA2 levels are ≤ 346 ng/ml compared to when they exceed 346 ng/ml (Huang et al., 2018). Soluble ST2 (sST2), as the soluble form of a member of the interleukin-1 receptor family, has been linked to adverse outcomes in various inflammatory and cardiovascular diseases when its levels are elevated (Zhang et al., 2021). A retrospective study by Wang et al. found that in male patients with septic cardiomyopathy, sST2 levels are independently associated with 28-day mortality (HR 1.003; 95% CI, 1.002 - 1.003). Moreover, sST2 can reflect cardiac function, with its concentration positively correlated with the diastolic function index E/e′ ratio (p<0.001) and negatively correlated with the right ventricular systolic function index tricuspid annular plane systolic excursion (TAPSE) (p<0.001) (Wang et al., 2024). In sepsis patients presenting with myocardial dysfunction, serum levels of lipocalin 10 (Lcn10) are often elevated at admission. Wang et al. conducted a single-center observational pilot study involving 75 patients, which confirmed that elevated serum Lcn10 levels at admission are positively correlated with the 28-day mortality rate of sepsis patients. They also determined the optimal cutoff value for predicting death to be 2.664 ng/mL, with an AUC of 0.797, a 95% CI of 0.696 - 0.897, and P<0.001 (Wang et al., 2021). To date, research integrating these biomarker tests with scoring systems remains limited. Owing to the constraints of the original data from the MIMIC dataset, combining these biomarkers with scoring systems for comprehensive analysis remains a challenge. This represents a promising avenue for future research endeavors.

Despite their reliability in assessing cardiac function, BNP and troponin perform poorly in evaluating SIMI. Their release is influenced by multiple factors, including systemic inflammation and renal insufficiency. Moreover, laboratory testing for these biomarkers is time-consuming and cannot provide immediate results. In light of these limitations, the 2023 ESC guidelines for sepsis management emphasized that single biomarkers are insufficient for prognostic assessment in SIMI due to their limited sensitivity and specificity. There is an urgent need to integrate multidimensional indicators to establish an effective risk prediction model that can more accurately reflect the dynamic nature of the disease and guide clinical decision-making (Guarino et al., 2023).

To facilitate early prognosis assessment of SIMI, there is an urgent need for a standardized evaluation criterion to assist clinical decision-making. Classic scoring systems, such as SOFA, APSIII, SIRS, LODS, OASIS, CHARLSON, GCS, and MELD, may hold some predictive value. These systems have been widely used in clinical practice to assess the severity of illness and predict outcomes in patients with various critical conditions.

SIRS score has been a widely used tool for identifying systemic inflammation. While it was highly sensitive (0.85) in detecting sepsis, it fell short in terms of specificity (0.41) and was less effective in predicting sepsis outcomes (Qiu et al., 2023; Usman et al., 2019) comprehensive system that quantifies dysfunction across six major organ systems to produce a total score. It demonstrated good predictive value for 30-day mortality in sepsis patients (AUC 0.73) and offers some clinical net benefit (Tekin et al., 2024). However, when compared with the more concise and commonly employed SOFA score, LODS did not show a significant advantage in predicting in-hospital mortality (Seymour et al., 2016). OASIS score evaluates illness severity by incorporating factors such as pre-ICU hospital stay duration, age, GCS, heart rate, mean arterial pressure, respiratory rate, temperature, urine output, ventilation status, and elective surgery. OASIS was significantly associated with in-hospital mortality in sepsis patients (OR = 1.07, 95% CI [1.06–1.08]) (Chen et al., 2019). CHARLSON score assesses the risk of adverse events by evaluating chronic diseases and comorbidities. It served as a predictor of mortality in sepsis patients (OR = 1.59, 95% CI 1.31–1.93) (Oltean et al., 2012). GCS evaluates a patient’s level of consciousness through assessments of eye-opening, verbal, and motor responses. It is incorporated into various scoring systems, including APACHE II, LODS, and SOFA, to assess neurological function. A prospective cohort study by Meral et al. (2024) validated that GCS can predict mortality in critically ill emergency patients, with an AUC of 0.65 for infected patients and 0.67 for non-infected patients. The MELD score, which includes creatinine, bilirubin, and the INR, is used to assess the prognosis of sepsis patients with liver dysfunction (Lin et al., 2024).

While the predictive capabilities of these scoring systems have been well-established in the context of sepsis, their potential for predicting SIMI remains to be further substantiated. SOFA score is a widely used tool for assessing organ dysfunction in sepsis patients and predicting their prognosis (Seymour et al., 2016), Moreover, the cardiovascular subsystem of the SOFA score (such as the demand for vasoactive drugs) directly quantifies sepsis-related hemodynamic disorders, which are closely related to the microcirculatory disorders and decreased cardiac output of SIMI. Research has shown that for every 1-point increase in the cardiovascular SOFA score, the risk of death in sepsis patients rose by 18% (P<0.001) (Ko et al., 2023). As a dynamic monitoring tool, it outperformed the cardiovascular part of the MODS score in predicting mortality risk, with an AUC of 0.73 (Minne et al., 2008; Peres Bota et al., 2002), this suggested that it may have potential value in predicting SIMI, although further validation was needed. APSIII, a component of the APACHE II score, integrates multiple physiological indicators, including heart rate, blood pressure, oxygenation index, and lactate levels. It facilitated the early detection of occult shock in SIMI patients and the prediction of their mortality risk (Yuniar et al., 2023). A cohort study of ICU patients with heart failure revealed that APSIII significantly enhanced its predictive ability in patients with sepsis, with an AUC of 0.72 compared to 0.67 for other scores. This improvement could be related to the mechanism of interaction between multiple organs and expands the application boundary of APS III in the field of cardiovascular diseases (Yang et al., 2022). By incorporating multiple physiological indicators, APSIII provides a more comprehensive reflection of a patient’s condition, suggesting potential application value in the early prediction of SIMI.

To establish an early prognostic prediction system for SIMI, we analyzed data from the MIMIC-IV database. Logistic regression and Cox proportional hazards analyses identified the SOFA and APSIII scores as significant predictors of mortality in SIMI patients. ROC curve analysis further revealed that both SOFA and APSIII demonstrated good sensitivity in assessing mortality risk, with AUC values of 0.685 and 0.683, respectively. Optimal cutoff values were established at 10.5 for SOFA and 61.5 for APSIII. Kaplan-Meier analysis indicated that patients in the lower score groups had significantly better prognoses compared to those in the higher score groups. Decision curve analysis (DCA) confirmed that both SOFA and APSIII provided substantial net benefit in predicting mortality. Additionally, our study found that age and lactate levels were predictive of 28-day all-cause mortality in SIMI patients, findings that were consistent with those of Huang (Huang et al., 2023) and Song (Song et al., 2020). Additionally, in this study, both multivariable Logistic regression and Cox regression analyses indicated that diabetes was associated with a HR of 0.747 (95% CI, 0.641–0.871) and an OR of 0.677 (95% CI, 0.546–0.839) (**Supplementary Figure S1**). These findings may be closely related to patients’ blood glucose levels and the use of medications such as SGLT2 inhibitors, DPP-4 inhibitors, and metformin. (Han et al., 2023; Lin and Lin, 2016; Zhang et al., 2019; Fan et al., 2024) However, this statistical analysis should be interpreted with caution and needs to be further explored in future studies.

## Conclusion

SIMI is a critical component of sepsis-related multi-organ dysfunction, and its prognostic assessment is confronted with two major challenges. First, traditional echocardiographic parameters, such as left ventricular ejection fraction (LVEF), have a weak correlation with mortality because they fail to capture dynamic compensatory mechanisms. Second, single biomarkers, such as BNP and cTnI, are limited by systemic inflammation, resulting in inadequate sensitivity and specificity. In this study, we conducted a systematic evaluation of eight critical illness scoring systems using data from the MIMIC-IV database. Our findings demonstrated that the SOFA and APSIII scores independently predict 28-day mortality risk in SIMI patients. These scoring systems are user-friendly and can promptly reflect changes in patient condition, providing a valuable tool for rapid risk stratification at bedside. This discovery holds significant clinical value in identifying high-risk SIMI patients and guiding early interventions. Future research should aim to further validate these findings through prospective trials and explore the potential of integrating these scoring systems with other biomarkers and therapeutic interventions. Such efforts may enhance predictive accuracy and clinical benefits, ultimately improving patient outcomes.

### Limitations

This study has several limitations. First, its retrospective design inherently introduces biases. For instance, the lack of recorded echocardiographic data and information on the dosage and duration of vasopressor affected the accuracy of cardiac function assessment in SIMI patients. Second, the study did not evaluate the impact of therapeutic interventions, such as fluid resuscitation strategies, on the dynamic changes in scoring. Future research should incorporate these data to provide a more comprehensive assessment of how therapeutic interventions affect patient outcomes. Finally, although the influence of chronic heart disease was controlled through strict exclusion criteria, other comorbidities associated with SIMI, such as coronary atherosclerotic heart disease, heart failure, atrial fibrillation, or acute respiratory distress syndrome, were not fully explored. These comorbidities could significantly influence the prognosis of SIMI patients, and future studies should further investigate their underlying mechanisms and impact on outcomes.

## Data Availability

Publicly available datasets were analyzed in this study. This data can be found here: https://physionet.org/content/mimiciv/1.0/.
