# Supplementary material for: The predictive value of SOFA and APSIII scores for 28-day mortality risk in SIMI: a cohort study based on the MIMIC-IV database
Source: Front Cell Infect Microbiol. 2025 Jul 29;15:1574625. doi: 10.3389/fcimb.2025.1574625 (PMC12339570; doi:10.3389/fcimb.2025.1574625)
Supplement: Supplementary file 1 [file DataSheet1.docx]

To further elucidate the impact of diabetes on outcomes, we performed a survival analysis comparing 28-day all-cause mortality from sepsis-induced myocardial injury (SIMI) between diabetic and non-diabetic patients. The results indicated no significant difference in survival between these two groups (hazard ratio [HR] = 0.905, 95% confidence interval [CI] 0.789–1.038, p = 0.154). (Supplementary Figure 1)

Both multivariate logistic regression and Cox regression analyses indicated that diabetes may serve as a potential protective factor against mortality in SIMI. To further investigate this “protective” effect of diabetes, we incorporated mean blood glucose levels as a covariate and conducted additional logistic and Cox regression analyses. The results demonstrated that elevated mean blood glucose levels are independently associated with increased 28-day all-cause mortality in patients with SIMI (logistic regression: OR =1.002, 95% CI 1.000-1.003, p = 0.04; Cox regression: HR 1.003, 95% CI 1.002-1.004, p <0.001). This suggests that interventions aimed at glycemic control, including strict dietary management, lifestyle modifications, and pharmacological treatments, may contribute to improved outcomes in SIMI. Subsequently, we performed subgroup analyses, which revealed that insulin use is associated with a better prognosis in SIMI (HR = 1.45, 95% CI 1.14–1.84, p = 0.002). This finding further supports the notion that active glycemic control is beneficial for the prognosis of SIMI. Other factors, including medication use, are limited by the original data from the database and warrant further investigation. (Supplementary Figure 2)


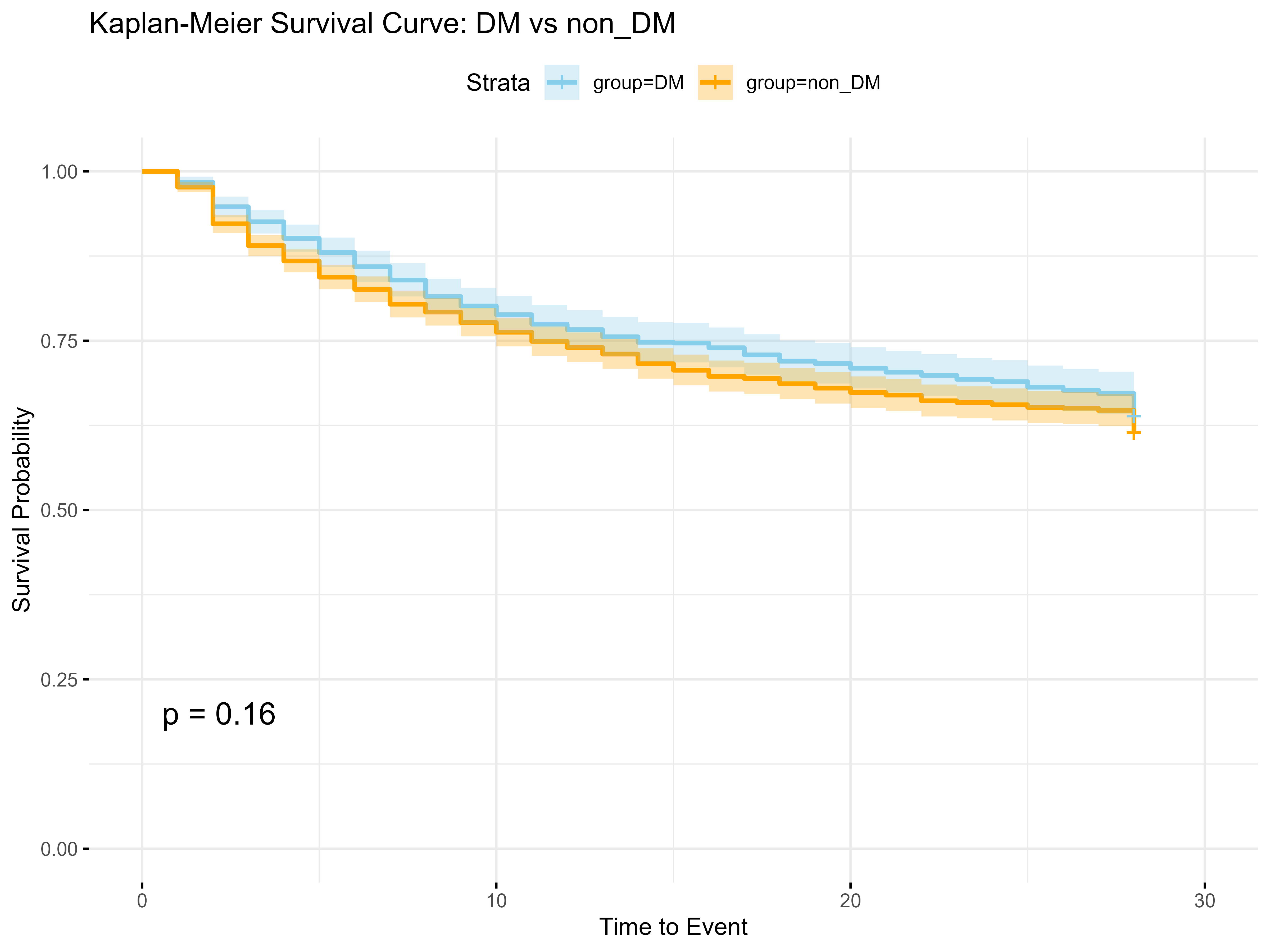


Supplementary Figure 1 Kaplan-Meier Survival Analysis for diabetes mellitus

DM: diabetes mellitus

**
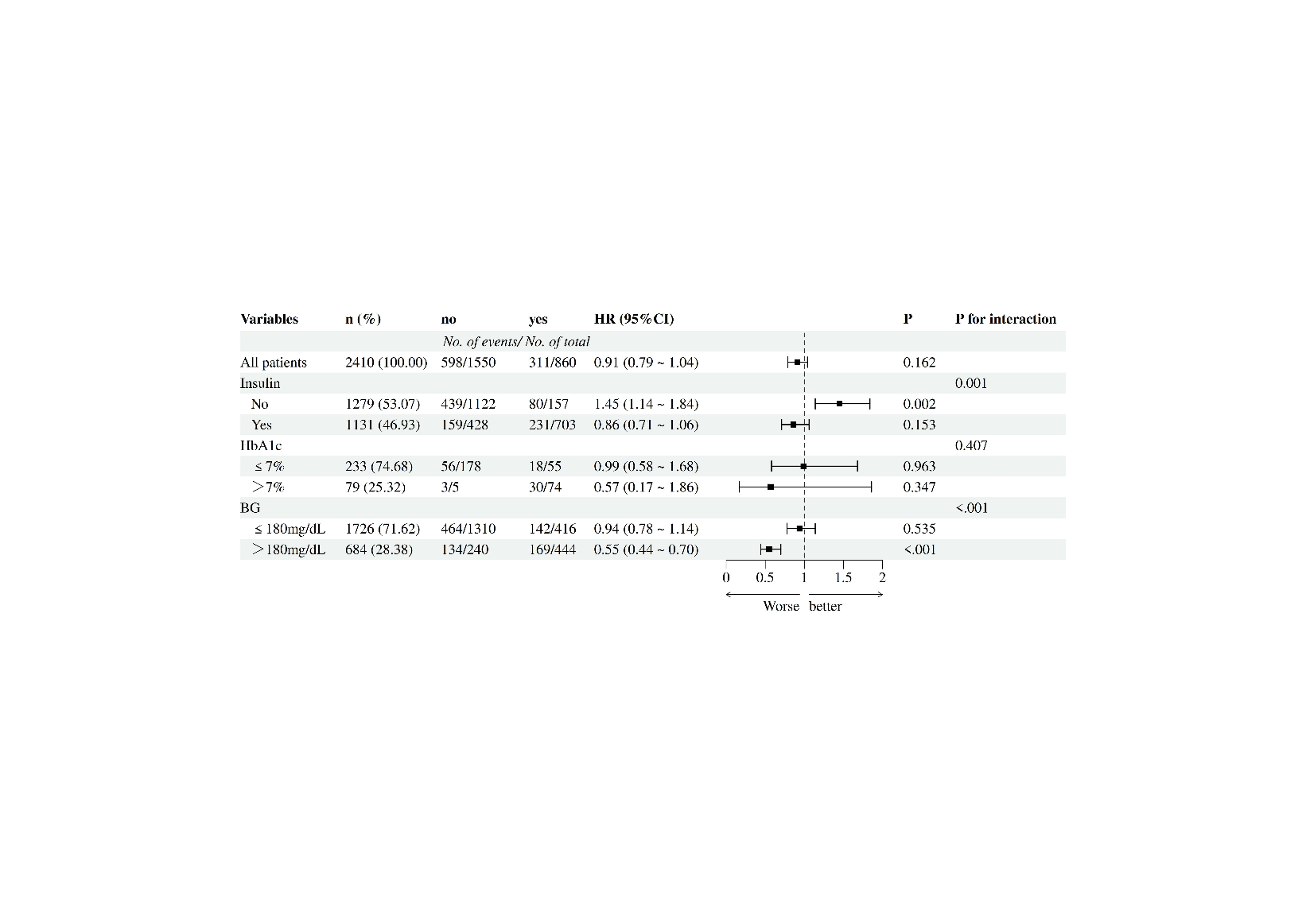
**

Supplementary Figure 2 Subgroup analysis of diabetes mellitus

BG: blood glucose
